# Thoracic chordoma in a 36-year-old female

**DOI:** 10.1093/jscr/rjac541

**Published:** 2022-12-05

**Authors:** Austin C Kassels, Joshua Melamed, Austin Rogers, David Johnstone

**Affiliations:** Department of Surgery, Medical College of Wisconsin, Milwaukee, WI, USA; Department of Surgery, Division of Cardiothoracic Surgery, Medical College of Wisconsin, Milwaukee, WI, USA; Department of Surgery, Division of Cardiothoracic Surgery, Medical College of Wisconsin, Milwaukee, WI, USA; Department of Surgery, Division of Cardiothoracic Surgery, Medical College of Wisconsin, Milwaukee, WI, USA

## Abstract

Chordomas are rare tumors that occur in the bones of the skull base and spine, affecting 1 in 1 000 000 people per year. Thoracic chordomas comprise just 1% of chordomas. A 36-year-old female underwent a right video-assisted thoracoscopic surgical resection for a cystic mass at the level of T2-3 which was well-circumscribed. Despite efforts to achieve an intact resection, there was tumor spillage due to friability, and it was taken off the bony vertebral body with no margin. The final pathologic diagnosis was chordoma. Thoracic chordomas are rare, slow-growing, recurring neoplasms that require proper preoperative diagnostic imaging and ideally preoperative trocar computed tomography-guided biopsy from a posterior approach if anatomic access is possible. They are prone to dissemination and sarcomatous differentiation. The surgical approaches for reported thoracic chordoma tumors vary due to their rarity and the variation in tumor location and presentation.

## INTRODUCTION

A chordoma is a bone tumor that displays notochordal differentiation from the persistent notochordal elements of embryonic development [1]. Chordomas are rare tumors that occur in the bones of the skull base and spine, affecting 1 in 1 000 000 people per year [2, 3]. They present in the sacrum, skull base or mobile spine, with a prevalence of 50, 30 and 20%, respectively [1]. Thoracic chordomas comprise just 1% of chordomas [4–6]. Negative prognostic factors include larger tumor size, positive surgical margins, microscopic tumor necrosis, Ki-67 > 5% and local recurrence [7]. Data have shown that the only factor associated with a disease-free state for >5 years is margin-free en bloc resection [5].

## CASE REPORT

**Figure 1:**
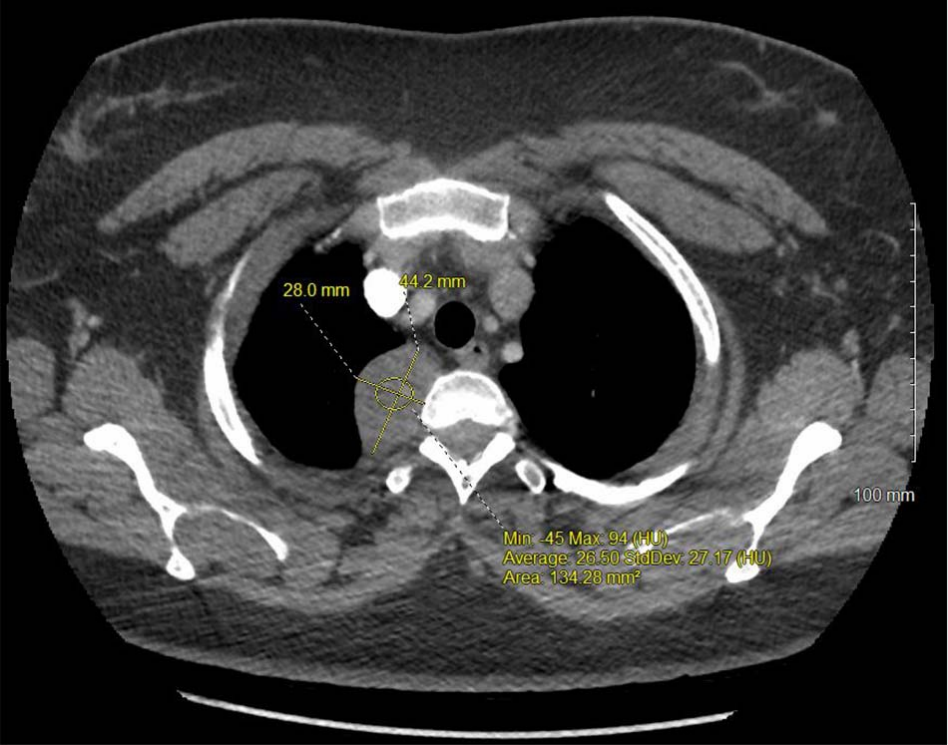
CT chest with IV contrast transverse view with detailed measurements of right T2-T3 paravertebral mass.

**Figure 2:**
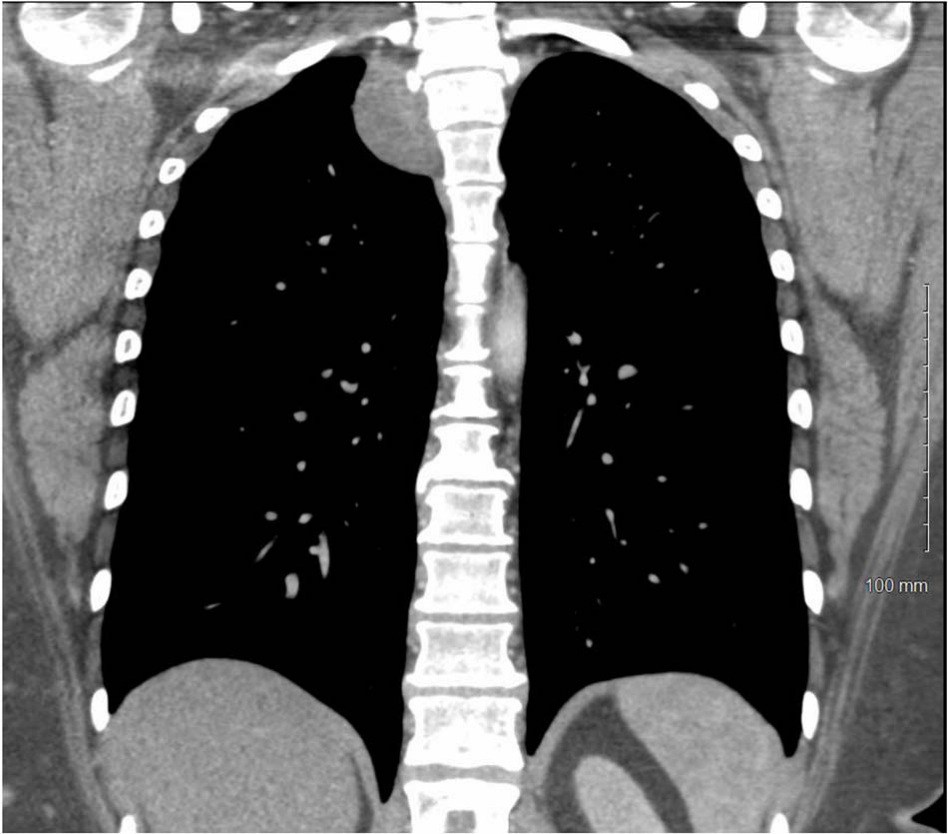
CT chest with IV contrast coronal view of right T2-T3 paravertebral mass.

A 36-year-old female presented to an outside emergency department reporting chest pain, dizziness and abdominal pain for 7 hours. A computed tomography (CT) angiogram of the chest revealed a right paraspinal 4.2 × 2.8 × 3.3-cm cystic mass at the level of T2-3, which was well-circumscribed (Figs 1 and 2).

**Figure 3:**
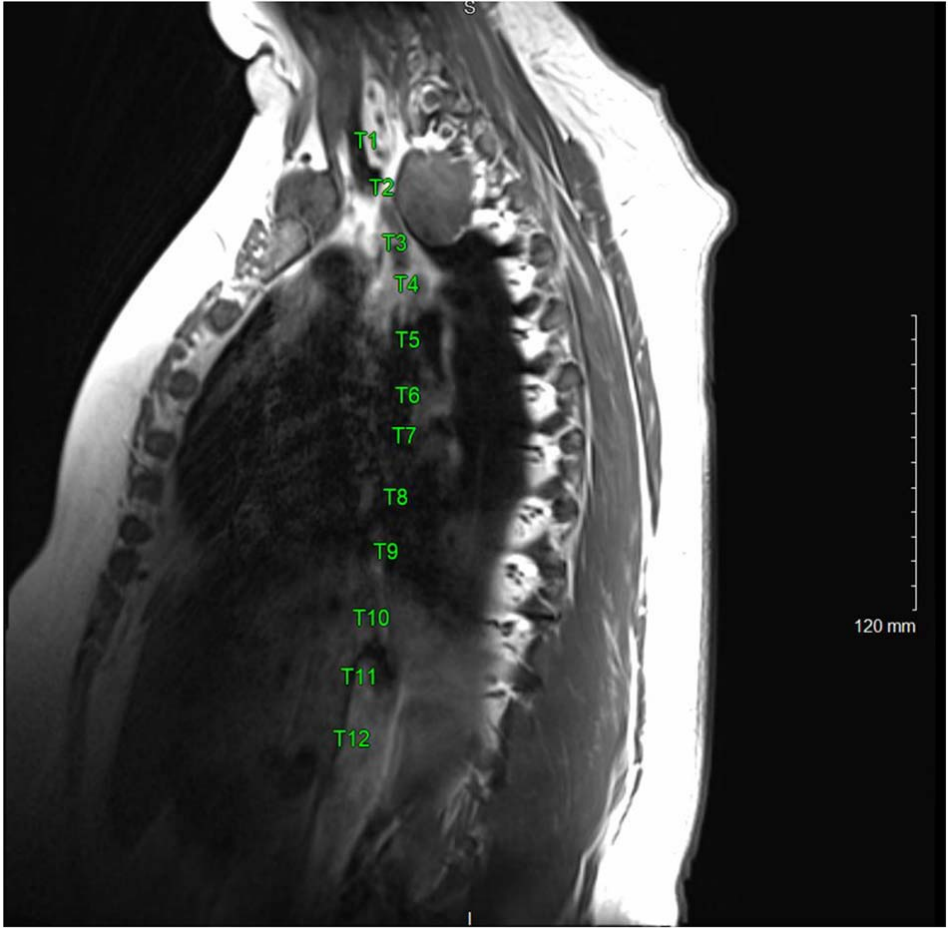
T1 MRI sagittal view of T2-T3 right paravertebral mass.

**Figure 4:**
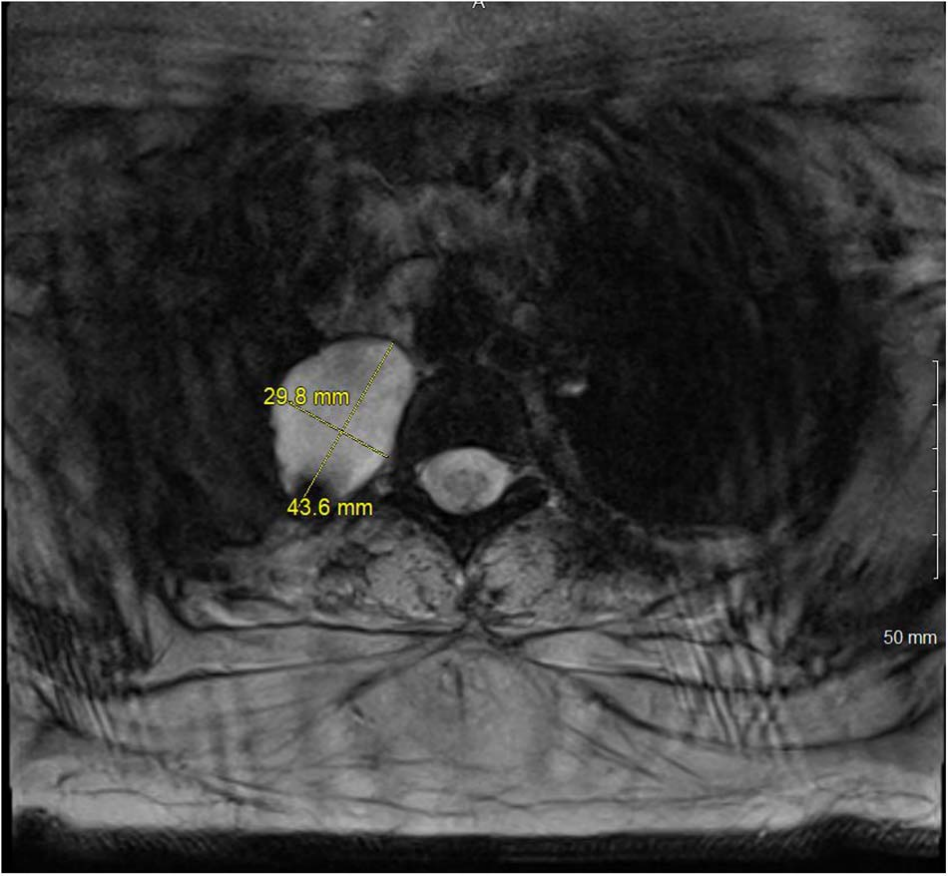
T2 MRI transverse view of T2-T3 paravertebral mass.

A magnetic resonance imaging (MRI) showed that the tumor extended into the outer aspect of the right T2-T3 neural foramen and could represent a nerve sheath tumor. Sixteen months later, neurosurgical consultation repeated her MRI, which showed that the mass had grown 2 mm (Figs 3 and 4). There was no extension into the neural foramen or bone erosion.

The patient underwent a right video-assisted thoracoscopic surgical resection. The mass was found to be gelatinous, not well-encapsulated and intimately involved with the sympathetic chain at the level of the first rib. Despite efforts to achieve an intact resection, there was tumor spillage due to friability, and it was taken off the bony vertebral body with no margin. The patient was discharged with a minor Horner’s syndrome which eventually resolved. The final pathologic diagnosis was chordoma. After multidisciplinary review, the patient was referred for proton beam therapy and received intensity-modulated radiation therapy to the tumor bed and then was referred for consideration of additional proton therapy.

## DISCUSSION

Thoracic chordomas are rare, slow-growing and recurring neoplasms that are prone to dissemination and sarcomatous differentiation [8]. Two publications, from 2012 and 2016, note finding ~30 reports since 1902 of thoracic spine chordomas [4, 9]. With regard to chordomas overall, most occur in middle age with a peak during the sixth decade of life [4–7]. Thoracic spine chordomas, however, present earlier with mean age at presentation of 35.7 years [4]. It is important to consider prognostic factors, such as age, sex, treatment history, tumor location, pathological grade, surgical margin, radiotherapy and chemotherapy, while determining long-term prognosis and considering treatment plans [10]. There is no effective systemic treatment of chordomas, and radiotherapy is combined with surgery to improve local control [10].

The surgical approaches for reported thoracic chordoma tumors vary due to their rarity and the variation in tumor location and presentation [4]. If possible, en bloc resection followed by proton beam therapy can be utilized as a treatment method to yield the longest disease-free period and highest rate of overall survival [4–6]. A major advantage of proton beam therapy over standard radiation therapy is that protons deposit the majority of their radiation dose directly in the tumor and do not travel further through the body. As a result, healthy tissues and organs receive less radiation [11].

The 5-year survival after surgical resection and radiation of spine chordomas vary between 50 and 60% [4–6]. The 10-year survival rate for chordomas is 40%, and the median overall survival for patients with these tumors is 7 years [12]. The Chordoma Consensus Group has classified surgical margins: wide resection with at least 1-mm margin, marginal resection with <1 mm around the tumor and intralesional resection with visible tumor left behind or tumor cells spilled into the surrounding area [13]. In this case, an intralesional resection occurred [5, 7].

Ideally, a preoperative tissue diagnosis would have been useful in this case. The preoperative findings were not specific. The preoperative presentation (constant, moderate and non-positional pain anteriorly in the right first and second intercostal area), while characteristic of a chordoma, may also be caused by other neurogenic lesions which are more common [14]. For spine tumors, a trocar CT-guided biopsy is recommended and should be done from a posterior approach [14]. Additionally, tissue samples should be evaluated for the presence of brachyury, which is expressed in high levels in chordomas [15]. A high index of suspicion would be required to pursue such workup, as these tumors often lack specific symptoms and present with non-specific findings on imaging [12].

Thoracic chordoma tumors are very rare tumors that require proper preoperative diagnostic imaging and ideally preoperative trocar CT-guided biopsy if anatomic access is possible. Non-specific symptoms and imaging characteristics, however, pose difficulties while developing differential diagnoses for cases involving thoracic chordomas because such a diagnosis is extremely rare. Ideally, chordomas should undergo margin-free en bloc resection to minimize recurrence. A combination of preoperative diagnostic results and intraoperative strategy should direct the excision of unknown thoracic tumors to be resected in the safest way possible to ensure complete resection and to prevent seeding during resection.

## AVAILABILITY OF MATERIALS AND DATA

As this paper is a case report, all data generated or analyzed are included in this article.

## References

[1] World Health Organization, WHO . In: Fletcher C, Bridge JA, PCW H, Mertens F (eds). WHO Classification of Tumours of Soft Tissue and Bone: WHO Classification of Tumours, Vol. 5, 4th edn. Geneva: World Health Organization, 2013, 328–9.

[2] Newton HC . In: Raghavan D, Blanke CD, Johnson DH, Moots PL, Reaman GH, Rose PG, et al. (eds). Textbook of Uncommon Cancer. Hoboken: John Wiley & Sons, Inc, 2012, 721–32.

[3] Stacchiotti S, Casali PG, Lo Vullo S, Mariani L, Palassini E, Mercuri M, et al. Chordoma of the mobile spine and sacrum: a retrospective analysis of a series of patients surgically treated at two referral centers. Ann Surg Oncol 2010;17:211–9. 10.1245/s10434-009-0740-x 19847568

[4] Fontes R, O'Toole JE. Chordoma of the thoracic spine in an 89-year-old. Eur Spine J 2012;21:S428–32. 21866405 10.1007/s00586-011-1980-6 PMC3369041

[5] Boriani S, Bandiera S, Biagini R, Bacchini P, Boriani L, Cappuccio M, et al. Chordoma of the mobile spine: fifty years of experience. Spine (Phila Pa 1976) 2006;31:493–503. 16481964 10.1097/01.brs.0000200038.30869.27

[6] Bjornsson J, Wold LE, Ebersold MJ, Laws ER. Chordoma of the mobile spine. A clinicopathologic analysis of 40 patients. Cancer 1993;71:735–40. 8431853 10.1002/1097-0142(19930201)71:3<735::aid-cncr2820710314>3.0.co;2-8

[7] Bergh P, Kindblom LG, Gunterberg B, Remotti F, Ryd W, Meis-Kindblom JM. Prognostic factors in chordoma of the sacrum and mobile spine: a study of 39 patients. Cancer 2000;88:2122–34. 10813725 10.1002/(sici)1097-0142(20000501)88:9<2122::aid-cncr19>3.0.co;2-1

[8] Topsakal C, Bulut S, Erol FS, Ozercan I, Yildirim H. Chordoma of the thoracic spine--case report. Neurol Med Chir (Tokyo) 2002;42:175–80. 12013671 10.2176/nmc.42.175

[9] Goomany A, Timothy J, Robson C, Rao A. En bloc resection of a thoracic chordoma is possible using minimally invasive anterior access: an 8-year follow-up. J Neurosci Rural Pract 2016;7:138–40. 26933363 10.4103/0976-3147.172171 PMC4750314

[10] Bai R, Zhao ZQ, Wang YX, Zhao W, Wu LS, Cui SX, et al. Sacral and thoracic chordoma with pulmonary metastases: a case report and review of the literature. Mol Clin Oncol 2021;14:17. 33363727 10.3892/mco.2020.2179 PMC7725214

[11] Science of Proton Therapy . How It Works - NAPT. NAPT. Published, 2018, https://www.proton-therapy.org/science/ (4 August 2022, date last accessed).

[12] Burke JF, Chan AK, Mayer RR, Garcia JH, Pennicooke B, Mann M, et al. Clamshell thoracotomy for en bloc resection of a 3-level thoracic chordoma: technical note and operative video. Neurosurg Focus 2020;49:E16. 10.3171/2020.6.FOCUS20382 32871571

[13] Stacchiotti S, Gronchi A, Fossati P, Akiyama T, Alapetite C, Bauman M, et al. Best practices for the management of local-regional recurrent chordoma: a position paper by the Chordoma Global Consensus Group. Ann Oncol 2017;28:1230–42. 28184416 10.1093/annonc/mdx054 PMC5452071

[14] Chordoma Foundation. Diagnosing Chordoma. https://www.chordomafoundation.org/learn/diagnosing-chordoma/ (26 August 2022, date last accessed).

[15] Sheppard HE, Dall'Agnese A, Park WD, Shamim MH, Dubrulle J, Johnson HL, et al. Targeted brachyury degradation disrupts a highly specific autoregulatory program controlling chordoma cell identity. Cell Rep Med 2021;2:100188. 33521702 10.1016/j.xcrm.2020.100188 PMC7817874

